# Modified IDSA/ATS minor criteria for severe community-acquired pneumonia best predicted mortality: Notification

**DOI:** 10.1097/MD.0000000000016914

**Published:** 2019-08-16

The Editorial Office of Medicine would like to acknowledge the Notice of Overlapping Publication published by *The American Journal of the Medical Sciences*, based on the article “Priority for Treatment and Intensive Care of Patients With Non-Severe Community-Acquired Pneumonia”^[1]^ and the *Medicine* article, “Modified IDSA/ATS Minor Criteria for Severe Community-Acquired Pneumonia Best Predicted Mortality”.^[2]^

The database used in this manuscript overlapped or partly overlapped with databases used in other published articles.^[3,4,5]^ While each of these studies tested distinct questions, the authors failed to disclose the repeated use of the database. The authors would like to issue an apology to the readers.
